# A loss-of-function variant in *ZCWPW1* causes human male infertility with sperm head defect and high DNA fragmentation

**DOI:** 10.1186/s12978-024-01746-9

**Published:** 2024-02-03

**Authors:** Yuelin Song, Juncen Guo, Yanling Zhou, Xingjian Wei, Jianlan Li, Guohui Zhang, Hongjing Wang

**Affiliations:** 1 Department of Obstetrics and Gynaecology, West China Second University Hospital, Sichuan University, Chengdu, 610041 China; 2 Key Laboratory of Birth Defects and Related Diseases of Women and Children of the Ministry of Education, West China Second University Hospital, Sichuan University, Chengdu, 610041 China; 3 SCU–CUHK Joint Laboratory for Reproductive Medicine, West China Second University Hospital, Sichuan University, Chengdu, 610041 Sichuan China; 4 Department of Obstetrics and Gynaecology, Southwest Medical University, Luzhou, 646000 China; 5 Child Healthcare Department, Sichuan Provincial Maternity and Child Health Care Hospital, Chengdu, 610000 China; 6 Chengdu University of Traditional Chinese Medicine, Chengdu, 611137 China; 7 Key Laboratory of Reproductive Medicine, Sichuan Provincial Maternity and Child Health Care Hospital, Chengdu, 610000 China

**Keywords:** *ZCWPW1*, Gene mutation, Male infertility, DNA fragmentation, DSB repair

## Abstract

**Background:**

Male infertility is a global health issue. The more causative genes related to human male infertility should be further explored. The essential role of Zcwpw1 in male mouse fertility has been established and the role of ZCWPW1 in human reproduction needs further investigation to verify.

**Methods:**

An infertile man with oligoasthenoteratozoospermia phenotype and his parents were recruited from West China Second University Hospital, Sichuan University. A total of 200 healthy Han Chinese volunteers without any evidence of infertility were recruited as normal controls, while an additional 150 infertile individuals were included to assess the prevalence of *ZCWPW1* variants in a sporadic male sterile population. The causative gene variant was identified by Whole-exome sequencing and Sanger sequencing. The phenotype of the oligoasthenoteratozoospermia was determined by Papanicolaou staining, immunofluorescence staining and electron microscope. In-vitro experiments, western blot and in-silicon analysis were applied to assess the pathogenicity of the identified variant. Additionally, we examined the influence of the variant on the DNA fragmentation and DNA repair capability by Sperm Chromatin Dispersion and Neutral Comet Assay.

**Results:**

The proband exhibits a phenotype of oligoasthenoteratozoospermia, his spermatozoa show head defects by semen examination, Papanicolaou staining and electron microscope assays. Whole-exome sequencing and Sanger sequencing found the proband carries a homozygous *ZCWPW1* variant (c.1064C > T, p. P355L). Immunofluorescence analysis shows a significant decrease in ZCWPW1 expression in the proband’s sperm. By exogenous expression with *ZCWPW1* mutant plasmid in vitro, the obvious declined expression of ZCWPW1 with the mutation is validated in HEK293T. After being treated by hydroxyurea, MUT-ZCWPW1 transfected cells and empty vector transfected cells have a higher level of γ-H2AX, increased tail DNA and reduced H3K9ac level than WT-ZCWPW1 transfected cells. Furthermore, the Sperm Chromatin Dispersion assay revealed the proband’s spermatozoa have high DNA fragmentation.

**Conclusions:**

It is the first report that a novel homozygous missense mutation in *ZCWPW1* caused human male infertility with sperm head defects and high DNA fragmentation. This finding enriches the gene variant spectrum and etiology of oligoasthenoteratozoospermia.

**Supplementary Information:**

The online version contains supplementary material available at 10.1186/s12978-024-01746-9.

## Background

Infertility is a significant global health issue, affecting 8–12% of couples of reproductive age [1]. In approximately 20–30% of cases, the primary factor leading to infertility may be attributed completely to the male partner, while an additional 20% of cases involve a male factor as a contributing cause [2, 3]. However, male infertility is complex and heterogeneous with a multitude of causes and risk factors, approximately 4% are affected by genetic disorders [4, 5]. A recent systematic review and clinical validity assessment of male infertility genes revealed that 120 genes were associated with 104 male infertility phenotypes. Among these 120 genes, 47 were definitively, strongly, or moderately associated with defects in spermatogenesis, leading to a significant reduction in sperm count (oligozoospermia) or a complete absence of sperm (azoospermia), elevated occurrence of aberrant chromosome numbers (aneuploidy), and low sperm quality (asthenozoospermia or teratozoospermia) [5, 6]. Furthermore, when the simultaneous occurrence of oligozoospermia, asthenozoospermia, and teratozoospermia cannot be explained, it is referred to as idiopathic oligoasthenoteratozoospermia (OAT), which is classified as a severe form of male infertility [7]. However, only few studies have reported the genetic alterations associated with OAT [8, 9].

Zinc finger CW-type and PWWP domain containing 1 (ZCWPW1) represents a constituent of the CW domain-containing protein family, exhibiting expression within the human testis [10–12]. Recently, a study found that Zcwpw1 deficiency disrupted spermatogenesis in male mice [13]. During spermatogenesis, diploid progenitor cells undergo meiosis to generate haploid germ cells, ensuring genetic variety via homologous recombination [14]. And in this process, recombination is initiated at prophase I through the deliberate induction of double-strand breaks (DSBs) [15]. Therefore, the production of DSBs and subsequent repair processes are crucial components of homologous recombination. The study found that the absence of Zcwpw1 in male mice leads to a total inability to generate synapses during meiosis, resulting in incomplete repair of DSBs and lack of crossover formation and indicates that Zcwpw1 might play a role in DSBs and its subsequent repair finally leading to male infertility [13]. Subsequently, several studies have firmly established that Zcwpw1 exhibits localization to DSB hotspots by specific recognition of H3K4me3 and H3K36me3, which are histone modifications deposited by PRDM9 at recombination hotspots in mice, promoting DSB repair [13, 16–19]. A recent study also revealed that Zcwpw1 inhibits the removal of H3K9ac by histone deacetylases, thereby facilitating the accessibility of chromatin to recombination hotspots. This process potentially enhances the occurrence of homologous recombination during DSB repair in meiosis [16]. Moreover, overexpression of ZCWPW1 in human somatic cells enhances DSB repair through homologous recombination [16]. These researches identified that ZCWPW1 plays an essential role in the process of DSB repair. However, it is currently uncertain the role of ZCWPW1 in human spermatogenesis and its potential implications for male fertility.

In our study, we first report an infertile patient harboring a homozygous variant of *ZCWPW1*. This variant was strongly associated with the clinical presentation of OAT, accompanied by an elevated level of DNA fragmentation in spermatozoa. Identification of mutation of *ZCWPW1* in infertile males holds heightened significance considering the challenging complexity of sperm quality selection in assisted reproductive technology procedures for OAT.

## Methods

### Study participants

The study conducted in the West China Second University Hospital involved a 36-year-old Han Chinese man, who had been diagnosed with infertility for 2 years. His sperm phenotype abnormalities were diagnosed at least twice and verified by semen analysis according to the World Health Organization’s (WHO) laboratory manual for the examination and processing of human semen (2021) [20]. To serve as the population study’s normal controls, 200 unrelated Han Chinese males were selected from among volunteers who had fathered at least one offspring through natural fertilization. In addition, 150 infertile individuals were recruited to determine the frequency of *ZCWPW1* variants in a sporadic male sterile population by whole‑exome sequencing (WES). The man’s parents were also involved in the study and they have no consanguinity. Oral pharyngeal swabs genomic DNA of his parents were collected for genetic analysis. The obstructive azoospermia patients provided the testicular puncture samples as the normal controls. This study was conducted according to the tenets of the Declaration of Helsinki and approved by the Ethical Review Board of West China Second University Hospital, Sichuan University. Before sample collection, each subject signed a written informed consent.

### WES and Sanger sequencing

Whole-blood samples from the proband and 150 infertile individuals were collected for WES. The genomic DNA was extracted from these samples using the FitAmp Plasma/Serum DNA Isolation Kit (P-1004-2, Epigentek) [21]. The SureSelect Human All Exon V6 Kit (5190–8865, Agilent Technologies) was used for exon capture according to the manufacturer’s protocol and sequencing was performed on the Illumina HiSeq X system [22]. ANNOVAR was utilized for functional annotation [22–25], followed by filtering of data using 1000 Genomes Project, gnomAD, and ExAC [26–28]. SNVs, SIFT, PolyPhen-2, M-CAP and CADD were employed to predict the functional consequences of the retained nonsynonymous [29–33]. The candidate variants should have a population frequency of less than 1/1000 and a minor allele frequency of less than 0.05. And bioinformatics functional analysis of included variants should be consistent with being deleterious or potentially deleterious. Oral pharyngeal swabs of his parents were collected for genetic validation of recessive inheritance of the candidate variant. The genomic DNA from the oral pharyngeal swab was extracted by using a rapid genomic DNA extraction kit for oral/pharyngeal swabs (D310, Aidlab Biotechnologies). Sanger sequencing was used to validate the candidate pathogenic variant detected in the patient and both of his parents. PCR amplification was performed using the ProFlex PCR System (Thermo Fisher) and DNA sequencing of PCR products was conducted on a DNA sequencer (ABI377A, Applied Biosystems). The *ZCWPW1* variant identified by WES was verified by Sanger sequencing. The PCR primers were as follows:

F: 5ʹ TTGTGTTCTGTTCATCTAACCCCTT 3ʹ;

R: 5ʹ GCCTGACTCATCTACCCTACCCTCG 3ʹ.

### Papanicolaou staining

Semen samples were carefully spread onto slides, air-dried, and fixed with 95% (volume/volume) ethanol for no less than 15 min. Following this, the slides were treated to a graded series of ethanol concentrations (50%, 80%, 95%) and stained sequentially with Harris’s haematoxylin (nuclei staining), acidic ethanol (decolorization), G-6 orange stain (non-squamous cytoplasm staining), and EA-50 green stain (cytoplasm staining), following the guidelines outlined by the World Health Organization [20]. The stained semen smears were mounted using permount TM mounting medium (MM1411, MKBio), enabling the preservation of the smears for further analysis under a microscope.

### Electron microscopy

The fresh sperm samples were prepared for electron microscopy using both scanning electron microscopy (SEM) and transmission electron microscopy (TEM) assays.

For SEM, the samples were fixed onto slides using 2.5% glutaraldehyde (as a fixative, preserving the structure and form of samples) and refrigerated overnight at 4 °C. The slides were rinsed with 1 × PBS buffer three times. Then gradually dehydrated with an ethanol gradient (30, 50, 75, 95, and 100% ethanol), which gradually remove water from the sample while preserving its structure, preparing it for further processing and imaging. The samples were then dried by a CO2 critical-point dryer (drying samples while preserving their structure) and sputter-coated by an ionic sprayer meter (Eiko E-1020, Hitachi). Finally, the samples were observed using SEM (S-3400, Hitachi).

For TEM, samples were washed three times with SpermRinse™ (10,101, Vitrolife), fixed in 3% glutaraldehyde, phosphate-buffered to pH 7.4, and postfixed with 1% OsO4. The cells were embedded in Epon 812, and ultrathin sections were stained with uranyl acetate (negative staining, providing contrast to the sample and making it easier to visualize the fine details of the specimen) and lead citrate (positive staining, visualizing cellular organelles and other subcellular structures in samples). High-quality imaging of the samples was performed under a TEM (TECNAI G2 F20, Philips) with an accelerating voltage of 120 kV.

### Isolation of human spermatogenic cells

The obstructive azoospermia patients provided the testicular puncture samples as the normal controls. The cell density-gradient centrifugation technique was performed using the STA-PUT velocity sedimentation method, as previously described [34, 35]. Briefly, spermatogenic cells of humans extracted from testis biopsies undergone the first digestion using 2 mg/ml collagenase IV (17104019, Gibco) and 1 μg/μl DNase I (18047019, Invitrogen). Then secondly digested by 4 mg/ml collagenase IV, 2.5 mg/ml hyaluronidase (H3506, Sigma), 2 mg/ml trypsin (T1426, Sigma) and 1 μg/μl Dnase I. Last, the samples were loaded in an STA-PUT velocity sedimentation cell separator (ProScience) for gradient separation. This method efficiently separates sperm cell populations by density, enabling the isolation and analysis of specific cell types.

### Immunofluorescence staining

For the analysis of spermatogenic cells and sperm, samples were fixed in 4% paraformaldehyde for 30 min, permeabilized with 0.3% Triton X-100 (HFH10, Invitrogen) for 15 min, and blocked with 10% donkey serum (C2540-0100, VivaCell) for 1 h at room temperature. The samples were then incubated overnight at 4 °C with primary antibodies against ZCWPW1 (1:2000; 165445, NovoPro), α-Tubulin (1:10,000; AC012, ABclonal), Peanut agglutinin (PNA) (1:100; L32459, Invitrogen), followed by incubation with Alexa Fluor 488 (1:1000; A21206, Thermo Fisher Scientific) or Alexa Fluor 594 (1:1000; A11005, Thermo Fisher Scientific)-labeled secondary antibodies at room temperature. Nuclei were counterstained with 4′,6-diamidino-2-phenylindole (DAPI) (D9542, Sigma-Aldrich).

For testicular tissues, samples were fixed in 3.7% buffered formaldehyde and embedded in paraffin. After the tissue sections were deparaffinized in xylene and rehydrated in a graded series of alcohols, they were treated with 3% hydrogen peroxide and 20 mM sodium citrate. Then, they were blocked with donkey serum, incubated with primary antibodies overnight at 4 °C, and with secondary antibodies and DAPI for 1 h at 37 °C. Finally, images were captured using a confocal microscope (FV3000, Olympus Corporation). The relative intensity of immunofluorence was quantified with ImageJ software (NIH) and used for statistical analysis.

### Real-time polymerase chain reaction

The RNA of the several tissues of mouse was extracted from Trizol reagent (15596026, Invitrogen). The cDNA was obtained using a RevertAid First-Strand cDNA Synthesis Kit (K1621, Thermo Fisher Scentific) following the protocol. Real-time PCR was performed using the TB Green Premix Ex Taq II (CN830S, TaKaRa).

The primers for mouse *ZCWPW1* were as follows:

F: 5ʹ-GGAGGAGAAGGAGGAGGAAGAA-3ʹ, 

R: 5ʹ-CAGTGTGGGTACAGGAGGGACT-3ʹ;

The primers for mouse *Gapdh* were as follows:

F: 5ʹ-GGTGAAG GTCGGTGTGAACG-3ʹ,

R: 5ʹ-CTCGCTCCTGGAAGATGGTG-3ʹ.

### Western blotting

The proteins from the cultured cell were extracted with the RIPA Lysis buffer (P0013B, Beyotime) and proteinase inhibitor cocktail (04693132001, Roche). The protein was obtained from cell lysis after centrifugation of 15,000 × g for 15 min. Then added SDS sample loading buffer (P0015, Beyotime) and the samples were boiled at 95 °C for 10 min. The concentration of the proteins was measured by BCA kit (P0011, Beyotime) following the manufacturer’s instructions. After denaturation, 40 μg protein was loaded in each lane. Proteins were separated by sodium dodecyl sulfate–polyacrylamide gel electrophoresis and transferred to a polyvinylidene difluoride membrane (IPFL00010, Millipore). The membranes were incubated with the instant blocked buffer (SW3012, Solarbio) for 20 min. Then the membranes were incubated with the primary antibody, Flag antibody (1:10,000; PTM-6075, PTM Bio), γ-H2AX (1:500; AP0687, ABclonal), H3K4me3 (1:500; A22146, ABclonal), H3K36me3 (1:500; A20379, ABclonal) and H3K9ac (1:500; A7255, ABclonal) with proper dilutions overnight at 4 ℃. Next, the membrane was incubated with goat anti-mouse IgG secondary antibody-HRP (1:2000; 32230, Thermo Fisher Scientific) or goat anti-rabbit IgG secondary antibody-HRP (1:2000; 6120, Thermo Fisher Scientific) at room temperature for 1 h. Finally, the blots were soaked with enhanced chemiluminescence (32209, Thermo Fisher Scientific) and were captured by the Chemidoc MP Imaging System (Bio-Rad). The grayscale analysis of protein bands was quantified with ImageJ software (NIH) and used for statistical analysis.

### Cell culture and transfection

HEK293t and Hela cells were cultured with the DMEM supplemented with 4.5 g/L glucose (10569010, Gibco), 10% fetal bovine serum (12484010, Gibco) and 1% penicillin/streptomycin (15070063, Gibco). The HEK293T or Hela cells in 6-well plates were transfected with the *ZCWPW1*-WT-Flag plasmid, *ZCWPW1*-MUT-Flag plasmid and pCAG plasmid respectively using the jetPRIME Transfection Reagent (101000046, Polyplus) following the manufacturer’s protocol.

### Sperm chromatin dispersion (SCD) assay

Sperm chromatin dispersion was evaluated with fresh semen using the SCD assay following the procedure previously described [36] [37]. A minimum of 200 sperm were microscopically evaluated for each semen sample, and sperm containing fragmented DNA displayed very small or no halos. The sperm DNA fragments index (DFI%) is calculated by the number of sperms with DNA fragments / the total number of sperms counted × 100%.

### Neutral comet assay

The HEK293T cells were transfected with ZCWPW1-WT-Flag plasmid, ZCWPW1-MUT-Flag plasmid and pCAG plasmid. After 24 h, HEK293T cells were treated with culture medium added with 2 mM hydroxyurea (HY-B0313, MedChemExpress) for 12 h. The slides were coated with 80ul 0.8% normal-gelling agarose first. A total of 1 × 10^4^ cells in 10 μl PBS were mixed with 70 μl 0.8% low-gelling agarose and were layered on the first normal-gelling agarose layer. The slides were immersed in the neutral lysis buffer (2.5 M NaCl, 100 mM Na_2_EDTA, 10 mM Tris, 1% N-lauroylsarcosine, 1% TritonX-100) for 1 h. For the DNA unwinding procedure, the slides were incubated in fresh neutral electrophoresis buffer (300 mM sodium acetate, 100 mM Tris, PH = 8.3) for 20 min in the dark. The electrophoresis was performed in neutral electrophoresis buffer at 15 V and 80 mA for 30 min. The samples were counterstained with DAPI. The pictures were captured by the fluorescence microscope (AX70, Olympus).

## Results

### Presentation of morphological abnormalities of the sperm flagella symptoms in the proband

**Fig. 1:**
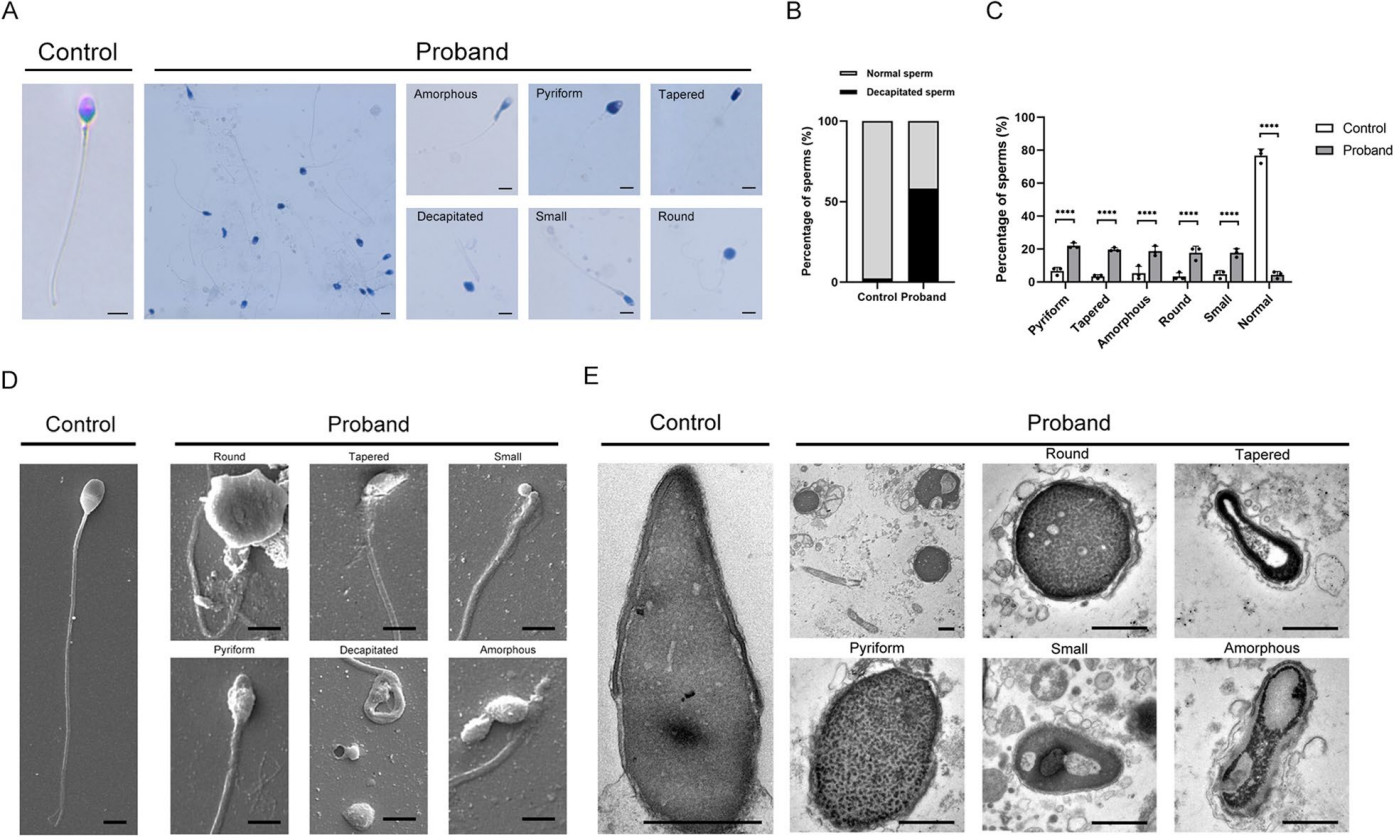
The anomalous sperm heads of various shapes in the patient spermatozoa. **A** Papanicolaou staining of patient sperms showed amorphous, pyriform, tapered, decapitated, small and round head (scale bars, 5 μm). **B** and **C** The distribution of deformed sperm heads in normal control and the infertile patient. Each rate was calculated by counting 100 sperms. **D** Scanning electron microscopy and transmission electron microscopy analysis of spermatozoa obtained from a control individual and the patient with the ZCWPW1 variant. The most of spermatozoa from the patient showed round, tapered, small, pyriform, decapitated and amorphous heads (scale bars, 5 μm)

A 36-year-old man with a 2-year medical history of infertility was recruited for our study. He has no history of genital infection, abnormal male sexual characteristics, or testicular trauma. His wife, of reproductive age, did not have any fertility-related problems. A semen analysis of this proband revealed that his symptoms are indicative of OAT (Table 1). The sperm quantity is lower than the normal range and sperm mobility is 18%, with the normal range being 32%. Importantly, most sperm cells of the patient have severe head defects. In order to examine detailed abnormalities of sperm morphology, Papanicolaou staining was performed. Compared with normal volunteers as a control, the result shows that the sperms of the patient exhibited a high rate of decapitated sperms and a mosaic of abnormal-shaped heads including pyriform head, tapered head, amorphous head, round head and small head (Fig. 1A and B). The incidence rates of various abnormalities in the head were analyzed statistically (Fig. 1C). The SEM and TEM were further used to observe the abnormal-shaped sperm head and high rate of decapitated sperms (Fig. 1D and E). In general, the patient’s infertility appears to be caused by pathological head shaping of sperm.

**Table 1:** Semen parameters of the patient and normal control

Semen parameters	Patient	Normal control	Normospermic parameters^**#**^
Sperm volume (mL)	3.8	4.5	≥ 1.5
PH	7.4	7.4	≥ 7.2
Sperm concentration (million/mL)	8.6	73.8	≥ 15
Total sperm count (million/ejaculate)	32.7	332.1	≥ 39
Progressive motility sperm (%)	18	81	≥ 32
Vitality (%)	82	85	≥ 58
Normal spermatozoa (%)^*^	1.0	77	≥ 4
Head defect (%)^*^	99	12	–
Tail defect (%)^*^	24.9	19.8	–

^#^Lower and upper reference limits are shown according to the World Health Organization standards (WHO, 2021)

*The calculations were performed including 200 sperm based on the WHO laboratory manual for the examination and processing of human semen

### Homozygous loss-of-function mutation in *ZCWPW1* was responsible for the patient’s infertility

**Fig. 2:**
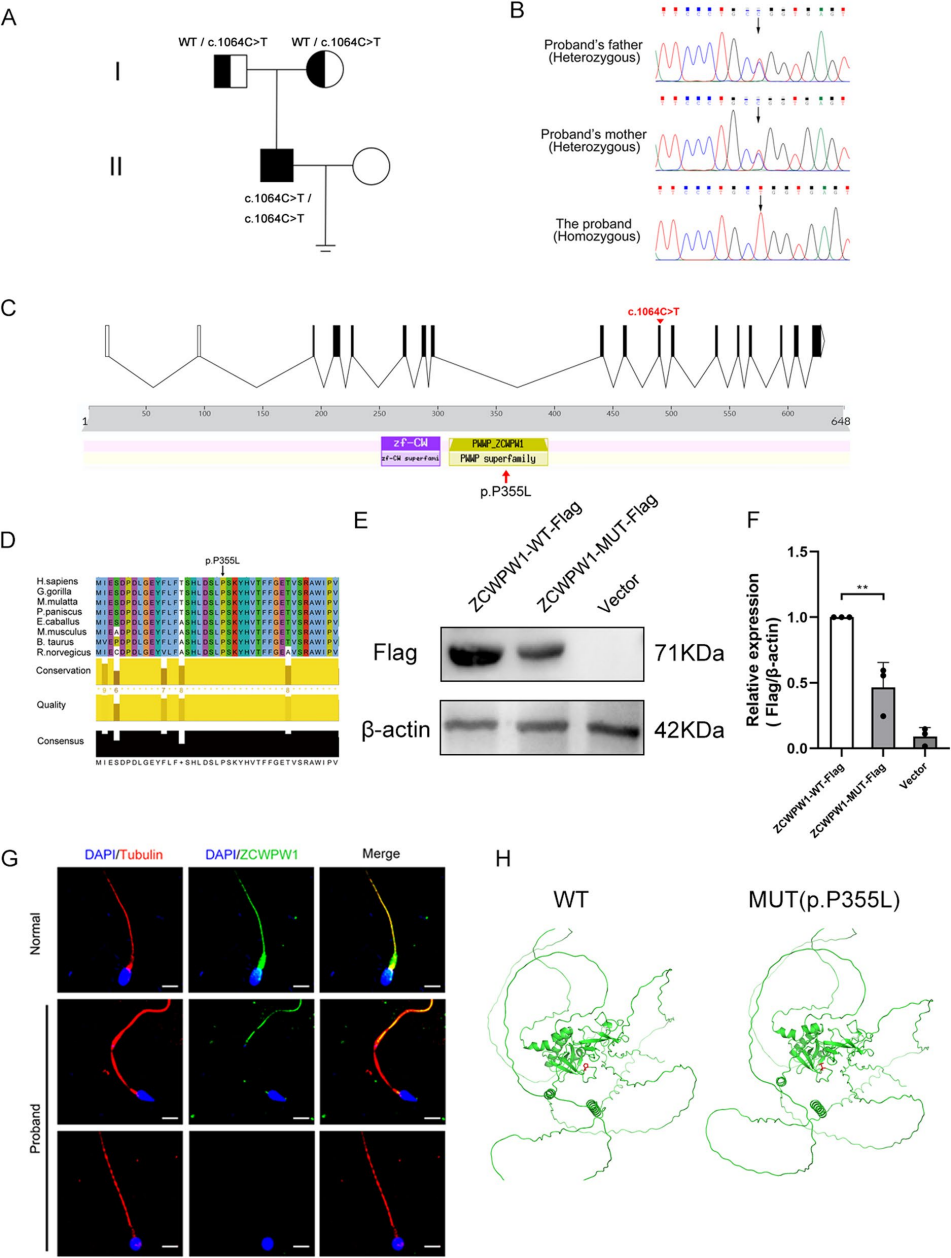
A missense homozygous variant in the *ZCWPW1* gene was identified in this infertile patient. **A** Family pedigree of the patient with *ZCWPW1* mutation. The proband is represented by the black square. **B** Sanger sequencing confirmed the mutation of c.1064C > T) in this family. The black arrow denotes the mutation position **C** The precise locations of the variant and changed amino acids in ZCWPW1. ZCWPW1 contains two domains: CW-TYPE zinc finger domain (amino acid positions 250–304) domain and PWWP (amino acid positions 317–383) domain. (D) Multiple sequence alignment of the ZCWPW1 protein for different species. The black arrow denotes the position of the variant (p. P355L). **E** Western blot reflected a marked decline in Mut-ZWCPW1 expression compared with WT-ZCWPW1. Vector is negative control. **F** The grayscale analysis of the protein bands was shown. Data represent the mean ± SD from three independent experiments. Student’s t-test, *P < 0.05, **P < 0.01. **G** The immunofluorescence staining showed the expression of ZCWPW1 in the spermatozoa from a fertile control individual and the patient. (Scale bars = 5 μm). **H** Effects of *ZCWPW1* mutation on the structural conformation of ZCWPW1 protein. Left panel is the structural conformation of ZCWPW1 protein wild type and right is the mutant. The amino acid positions are labeled in red. The structures of the ZCWPW1 protein were changed by the mutation of c.1064 C > T [p.P355L]

To identify the genetic cause of the OAT in this study, we performed whole-exome sequencing (WES) analysis on the proband. A homozygous missense mutation (c.1064C > T, p. P355L) was identified in *ZCWPW1* (NM_017984.6) (Table 2) based on the following inclusion criteria of potential variants. The allele frequencies in public databases (1000 Genomes Project, ExAC Browser, and gnomAD) were either not included or less than 1 ‰ [26–28]. Mutation prediction tools (SIFT, PolyPhen-2, M-CAP) predicted the mutation to be possibly detrimental or detrimental to protein function [29–33]. Additionally, the CADD tool indicated a score of 33 for the mutation, which is considered deleterious for scores above 30. Sanger sequencing of ZCWPW1 was performed in the proband’s family verifyng the heterozygous mutation in each parent and the homozygous mutation in the proband (Fig. 2A and B). The result of Sanger sequencing demonstrated that the infertility related to ZCWPW1 being inherited in an autosomal recessive manner. This mutation (c.1064C > T) is classified as a missense mutation and causes proline transform to leucine in the 355th amino acid position which is exactly in the PWWP domain of ZCWPW1 (Fig. 2C). In addition, the amino acid is highly conserved in most mammal species, from an evolutionary perspective, indicating its critical role in maintaining protein function (Fig. 2D).

**Table 2:** Details of ZCWPW1 variant carried by the patient

Gene	Mutation	Zygosity	Mutation Function	Variant Classification (ACMG)	Allele frequency			Functional prediction			
					1000G	ExAC	gnomAD	SIFT	PolyPhen-2	M-CAP	CADD
ZCWPW1NM_017984.6	rs200631169 c.1064C > T p. P355L	Homozygous	Missense	Pathogenic	NA	0.0001433	0.0001117	Probably deleterious	Deleterious	Possibly pathogenic	33

CADD: Hazard score, 10 points refer to top 10%, 20 refer to top 1%, 30 refer to top 0.1%; NA, not available

To further explore the effect of the missense mutation on the ZCWPW1 protein expression, ZCWPW1 -WT*-*Flag (Flag-tagged wild-type human *ZCWPW1*) and ZCWPW1 -MUT*-*Flag (Flag-tagged mutant human *ZCWPW1* with c.1064C > T) plasmid for expression were constructed. Then, the WT, MUT and empty vector plasmid were respectively transfected into HEK293T cells. The western blot results indicate a significant reduction in ZCWPW1-MUT-Flag expression when compared to ZCWPW1-WT-Flag (Fig. 2E and F). Furthermore, we stained ZCWPW1 in the transfected cells and found that the mutation did not alter the nucleus location of ZCWPW1 (Additional file 1: Fig. S1). Additionally, we used immunofluorescence and an anti-ZCWPW1 antibody to detect changes in ZCWPW1 expression on spermatozoa in the proband. Compared to the control, ZCWPW1 expression significantly reduced in proband’s sperm flagella (Fig. 2G). The analysis of relative fluorescence intensity of ZCWPW1 (Additional file 1: Fig. S2) indicates that the ZCWPW1 expression in the proband’s sperm has decreased by almost 50%. Thus, the homozygous mutation (c.1064C > T, p. P355L) leads to significantly reduced protein expression. To further understand the effect of this mutation, we investigated the conformational changes of the ZCWPW1 protein caused by these mutations with Phyre2 and PyMOL soft-ware41 (1.3r1, DeLano Scientific LLC) [38, 39]. The silico analysis revealed that the c.1064C > T mutation alters the ZCWPW1 protein conformation [p. P355L] (Fig. 2H). A proline to leucine mutation changes a rigid, cyclic residue to a more flexible, linear residue, potentially altering protein stability and folding. Overall, based on population data, computational analysis, functional criteria, and allelic and co-segregation data, the variant was classified as pathogenic by the American College of Medical Genetics and Genomics (ACMG) [40].

### ZCWPW1 robustly expresses in testes

**Fig. 3:**
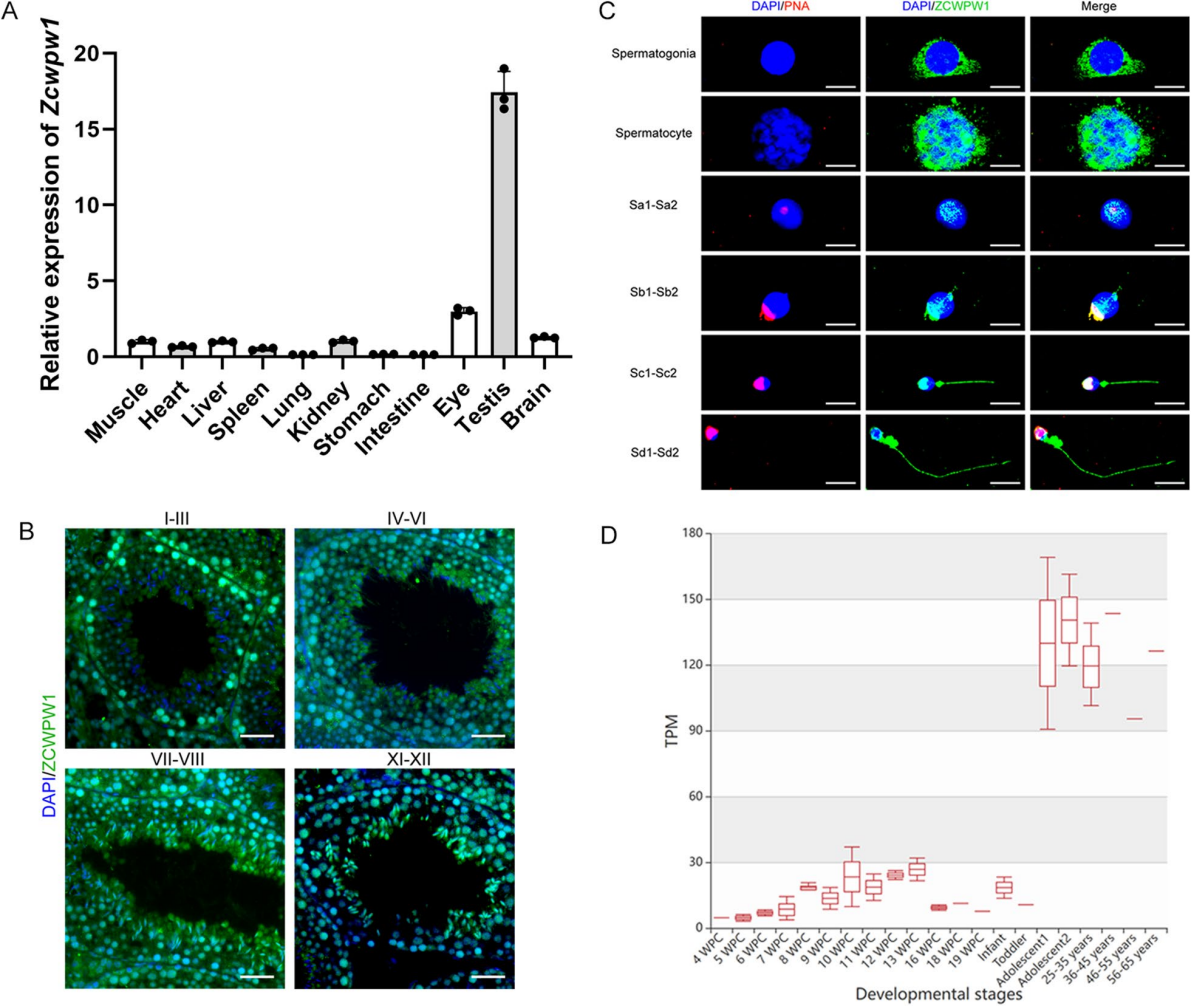
ZCWPW1 expression in mouse and human reproductive system. **A** Real-time polymerase chain reaction analysis (RT-PCR) revealed the expression of ZCWPW1 in the different mouse tissues. Quantification of the RT-PCR results by a histogram according to the cycle threshold value. **B** ZCWPW1 immunofluorescence staining for different stages of mouse spermatogenesis using mouse testis sections. (Scale bars, 50 μm). **C** Immunofluorescence staining for different stages of human spermatogenic cells (Scale bars, 5 μm). **D** The RNA expression levels of ZCWPW1 at different developmental stages in human testes (TPM = transcripts per million; WPC = weeks postconception)

To further understand the roles of ZCWPW1 in male reproduction, we first investigated the expression level of ZCWPW1 in different organs of adult mice. The results showed that *ZCWPW1* gene expression is enriched in mouse testes (Fig. 3A). ZCWPW1 is highly expressed in mouse testes, as seen through immunofluorescence analysis (Fig. 3B). This expression pattern could closely mirror that of human testes. Notably, we sorted the different stages of germ cells from human testes by STA-PUT velocity sedimentation. ZCWPW1 expression was detected in the cytoplasm of spermatogonia, nucleus and cytoplasm of spermatocytes at meiosis, acrosome of round spermatids and acrosome and tail of elongated spermatids (Fig. 3C). We speculated that the localization profile of ZCWPW1 during spermatogenesis is consistent with its known function. Prior to the initiation of meiosis in spermatogonia, the cells synthesize and accumulate ample amounts of ZCWPW1 protein in their cytoplasm. During the process of meiosis, the ZCWPW1 proteins accumulate in chromatin to facilitate the repair of DSBs. When germ cells transition into round spermatids, a portion of ZCWPW1 is disposed of along with excess cytoplasm. The remaining ZCWPW1 is stored in the acrosome. The presence of ZCWPW1 in both the nuclear and acrosome, from meiosis to elongated spermatids, is likely crucial for its regulatory role in sperm head formation. During spermatid elongation, ZCWPW1 is gradually transported to the tail region while the protein becomes temporarily inactive due to highly condensed DNA that prevents breakage. Our immunofluorescence staining results of human spermatogenic cells suggest that this transport accounts for the predominant signals of ZCWPW1 in sperm flagella with head defects (Fig. 2G). Then we explore the database to find out the RNA expression levels of *ZCWPW1* at different developmental stages in human testes (MeDas online repository). The results analysis shows that *ZCWPW1* is mainly expressed in testicular tissue of adolescents and adults (Fig. 3D). However, the role of *ZCWPW1* in the tail of spermatozoa is not yet fully understood and requires further investigation. In summary, these results suggest that ZCWPW1 is important for male reproduction in mice and humans.

### The mutation of *ZCWPW1* leads to insufficient capability of DSB repair resulting in high DFI in the sperm

**Fig. 4:**
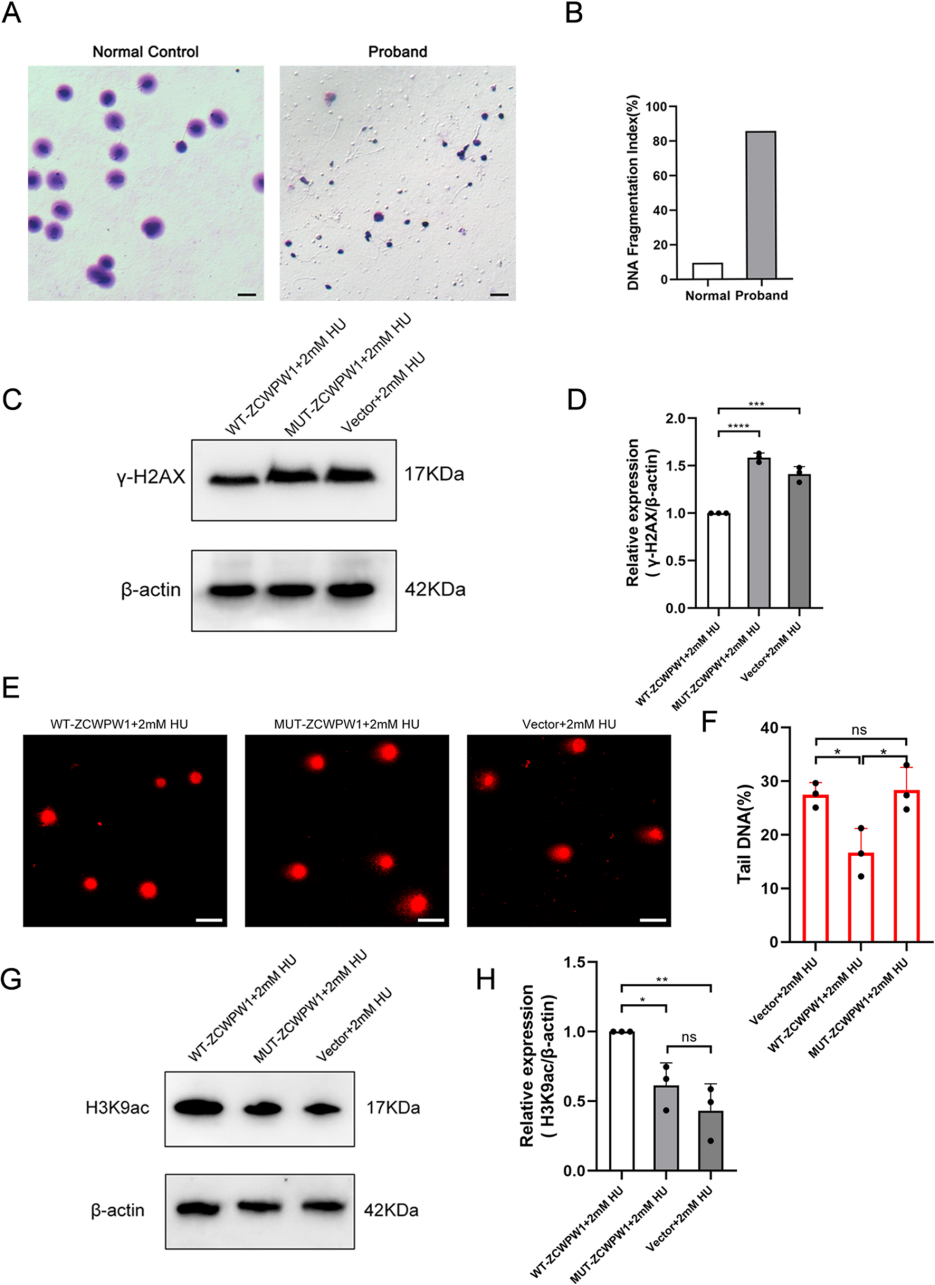
The homozygous mutation in *ZCWPW1* generated high DNA fragmentation and loss of the DSB repair capability. **A** The Sperm Chromatin Dispersion (SCD) analysis of spermatozoa obtained from a control individual and the proband by the optical microscopy (Scale bars, 20 μm). **B** The histogram showed the difference in DNA fragmentation index (DFI) between normal spermatozoa and proband’s spermatozoa. **C** The western blot showed the lower level of γ-H2AX in WT-ZCWPW1 transfected cells than MUT-ZCWPW1 transfected cells and empty vector transfected cells after treated by hydroxyurea for 12 h. 40 μg protein of extracts was loaded in each lane. **D** The grayscale analysis of γ-H2AX in each groups was shown. Data represent the mean ± SD from three independent experiments. Student’s t-test, ***P < 0.001, ****P < 0.0001. **E** The neutral comet assay found out the more DNA tail in MUT-ZCWPW1 transfected cells and empty vector transfected cells than WT-ZCWPW1 transfected cells after treated by hydroxyurea for 12 h (Scale bars, 10 μm). **F** Bar plot showing the ratio of tail DNA in the neutral comet assays. Data represent the mean ± SD from three independent experiments. Student’s t-test, *P < 0.05. **G** The western blot revealed the higher level of H3K9ac in WT-ZCWPW1 transfected cells than MUT-ZCWPW1 transfected cells and empty vector transfected cells after treated by hydroxyurea for 12 h. 40 μg protein of extracts was loaded in each lane. **H** Bar plot showing the grayscale analysis of H3K9ac. Data represent the mean ± SD from three independent experiments. Student’s t test, *P < 0.05, **P < 0.01

In our study, we discovered a homozygous mutation of *ZCWPW1* in the PWWP domain, which is crucial for maintaining its function. Previous structural studies have shown that PWWP domains contain a conserved aromatic cage specifically recognizing histone methyl-lysine [16, 41]. Moreover, these domains exhibit a cooperative binding affinity towards both histones and DNA, thereby contributing to their ability to bind nucleosomes and localize within chromatin [42]. Therefore, the mutation in the PWWP domain may disrupt the ZCWPW1 function of repairing DSBs. Upon observing that the mutation causes a decline in protein expression and structure alterations, we investigated its effect on DSB repair capability. We conducted a Sperm Chromatin Dispersion (SCD) assay to investigate the DFI in the patient’s sperm and normal controls (Fig. 4A and B). The percentage of patient sperm with DFI was significantly higher compared to normal controls (85.5% vs 9.5%). Additionally, there was a negative correlation between sperm DFI and sperm density, vitality, and normal morphology [43]. Therefore, the high DFI in the patient’s sperm may account for the patient’s defective semen parameters, including head defects, decapitated sperm, low quantity of sperm and low progressive motility. In vitro experiments, we exogenously expressed wildtype-ZCWPW1 and mutant ZCWPW1 in HEK293t cells. To assess the DSB repair capacity between wildtype-ZCWPW1, mutant ZCWPW1 and control (vector) cell, we used a DSB-inducing agent, hyroxyurea, to treat HEK293T cells, and measured γH2AX levels by western blot as an indicator of DSBs. Cells expressing wildtype ZCWPW1 has significantly lower levels of γH2AX compared to mutant ZCWPW1 cells and control cells, while the level of γH2AX in mutant ZCWPW1 cells is almost equivalent to that in control cells (Fig. 4C and D). In addition, after treating cells with hydroxyurea for 12 h, we conducted a neutral comet assay to assess the repair capability in mutant ZCWPW1. The tail DNA in wildtype-ZCWPW1 cells is found to be less abundant than in mutant ZCWPW1 cells and control cells. However, no significant difference in tail DNA is observed between the mutant ZCWPW1 cells and the control cells (Fig. 4E and F). These results indicated that wildtype-ZCWPW1 could enhance DSB repair but mutant ZCWPW1 nearly lost the DSB repair capability.

In previous reports, ZCWPW1 works to prevent histone deacetylases (HDACs) from removing H3K9ac, which keeps chromatin openness at recombination hotspots [16, 41]. It’s intriguing to note that when ZCWPW1 is expressed in human somatic cells, it can help with repairing DNA damage through homologous recombination. While the H3K9ac signal was significantly increased in HeLa cells expressing wildtype ZCWPW1 compared to control cells transfected with an empty vector, the H3K9ac signal in cells transfected mutant ZCWPW1 plasmid was comparable to that of control cells (Fig. 4G and H). This result revealed that the mutation of ZCWPW1 led to the failure of antagonistic function against HDACs. Taken together, we found the homozygous mutation in *ZCWPW1* results in protein loss of function, which may cause male infertility due to DSB repair deficiency in spermatogenesis.

## Discussion

In our study, it is the first report that mutation of *ZCWPW1* could cause male infertility in humans. We identified a novel homozygous missense mutation of c.1064C > T of *ZCWPW1* in an OAT patient, which resulted in reduced ZCWPW1 protein expression, loss of function of DSB repair, increased DNA fragmentation and reduced H3K9ac level, which probably lead to anomalies of sperm head morphology. Our research strongly suggests that this homozygous loss-of-function mutation in *ZCWPW1* is associated with male infertility.

ZCWPW1 is a reader of dual histone methylation, specific for obstructive azoospermia patient PRDM9-catalyzed histone marks H3K4me3 and H3K36me3, and maintaining H3K9ac level around recombination hotspots. It is an essential meiotic recombination factor required for the necessary repair of PRDM9-dependent DSBs [19, 44]. Li et al. reported that Zcwpw1 is essential for male fertility and spermatogenesis in mice [13]. They constructed the *Zcwpw1* knock-out mice and found the complete failure of synapsis resulting from the lack of DSB repair and crossover formation in the male mice testes. Impaired spermatogenesis was evident in *Zcwpw1* − / − male mice as compared to their *Zcwpw1* + / + counterparts. Notably, the seminiferous tubules of *Zcwpw1* − / − male mice exhibited an absence of post-meiotic spermatocytes while containing apoptotic cells. In our present study, our findings supported the pivotal role of ZCWPW1 in the spermatogenesis. Notably, we found the loss-of-function of ZCWPW1 in humans did not cause a completely similar phenotype with the *Zcwpw1 − / − *mice. Spermatozoa still could be produced in the proband’s testes, which meant the meiotic process of the proband was not completely disrupted. Whereas, according to the semen parameters of the proband, the quantity of sperm was lower than the normal threshold, indicating that the loss-of-function mutation in *ZCWPW1* remained to influence the normal process of meiosis. Furthermore, the evident phenotype of the proband was severe head defects of sperm, which was largely responsible for male infertility. There is a complex etiology for aberrations of the sperm head. On the one hand, plenty of causative genes involving in cytoskeletal related proteins were found in recent years. *DPY19L2* as the most frequent causative gene was reported to be associated with globozoospermia [45]. The DPY19L2 protein is found within the inner nuclear membrane of spermatozoa. Its function is to serve as an anchor, connecting the developing acrosome to the nuclear envelope through a specialized cytoskeletal plate called the acroplaxome [46]. It was revealed that spermatozoa spermatozoa from patients with mutations in the *DPY19L2* gene exhibited a separated inner nuclear membrane from the outer membrane and the entire detachment of the acrosome [47]. On the other hand, some new candidate genes involved in meiosis and epigenetic regulation, similar to *ZCWPW1* were found to account for sperm head abnormalities. *Meig1* was originally identified in mammalian potentially involved in meiosis. The expression of *Meig1* was increased through zygotene and pachytene stages. Zhang et al. generated a *Meig1*-conditional knockout mouse model and found the males were sterile and deformed sperm heads [48]. BRDT, as critical epigenetic readers binding to acetylated histones specifically in spermatocytes and spermatids, was reported to enriched in the patient with acephalic spermatozoa from a consanguineous family. The phenotype of *BRDT* mutated patients is similar to our proband with *ZCWPW1* mutation. *BRDT*-Knock-out mice exhibited infertility with complete absence of post-meiotic cells. The mechanism underlying occurrence of different phenotype between the patient and knock-out mice should be further investigated. Moreover, the loss function of PHD finger protein 7 (PHF7), a conserved epigenetic reader, leads to abnormal structure of sperm heads in mutant mice. Taken together, these evidences can shed light on the physiopathology of ZCWPW1 mutation in the patient and help us better understand the relationship between meiosis /epigenetic factors and sperm head formation.

Considering that the *ZCWPW1* mutation induced the increase in sperm DNA fragmentation, the causal relationship between high DFI and sperm head defects is still unclear. Two independent groups performed cross-sectional studies and revealed that high sperm DNA fragmentation was associated with sperm head defects [49, 50]. Additionally, Boe-Hansen et al. use sperm chromatin structure assay (SCSA) to detect the DFI in bulls. They concluded the percentage of morphologically normal spermatozoa was correlated negatively to DFI [51]. Wang et.al reported two mutations of *SEPT14* in the patient caused severely malformed sperm heads with high DNA fragmentation [52]. During the acrosome phase of spermiogenesis, the developing sperm head contains the acrosome and a condensing nucleus, while the axoneme grows to become the tail. Thus, while DNA fragmentation index (DFI) may be associated with abnormalities in the sperm head, it is unlikely to affect the neck-tail region. Therefore, these observations may help to explain the loss-of-function of ZCWPW1 in DSB repair in the spermatogenesis leads to sperm head deformation.

## Conclusions

Our study has identified a new variant of the *ZCWPW1* gene in a male patient who has sperm head defects and high DNA fragmentation. This is the first ever report of *ZCWPW1* gene in human male infertility, and it provides valuable information for genetic counseling and diagnosis for OAT. In order to better understand its role in male infertility, it is worthwhile to explore the interaction of ZCWPW1 with other related genes and signaling pathways which are crucial for reproductive development. The novel models such as testicular organoids and iPSC technologies are effective in-vivo platforms to investigate the pathological spermatogenesis process and clinical treatments. Further researches are required to investigate the impact of *ZCWPW1* mutation on the outcome of ART for affected individuals.

### Supplementary Information

**Additional file 1. Figure S1.** The localization of mutant ZCWPW1 did not alter in vitro. We used anti-Flag antibofy to detected exogenous ZCWPW1 in MUT-ZCWPW1 transfected cells compared to WT-ZCWPW1 transfected cells. Effectively transfected cells were marked with arrowhead. (Scale bars, 10μm). **Figure S2.** The reduced expression of ZCWPW1 in proband’s sperms. The relative fluorescence intensity analysis normalized by α-Tubulin was shown. Data represent the mean ± SD from three independent experiments. Student’s t-test, **P < 0.01.

## Data Availability

All data generated or analysed during this study are included in this published article.

## Abbreviations

ARTs: Assisted reproductive technologies
DSBs: Double-strand breaks
PRDM9: PR domain zinc finger protein 9
H3K4me3: Tri-methylation of lysine 4 of Histone 3
H3K36me3: Tri-methylation of lysine 36 of Histone 3
H3K9ac: Acetylation of lysine 9 of Histone 3
ZCWPW1: Zinc finger CW-type and PWWP domain containing 1
WHO: World Health Organization
SEM: Scanning electron microscopy
TEM: Transmission electron microscopy
DAPI: 4′,6-Diamidino-2-phenylindole
ACMG: American College of Medical Genetics and Genomics
DFI%: The rate of sperm DNA fragments
*ZCWPW1* -WT*-Flag*: Flag-tagged wild-type human *ZCWPW1*
*ZCWPW1* -MUT*-Flag*: Flag-tagged mutant *human ZCWPW1* with c.1064C > T
SCD: Sperm chromatin dispersion
