# Supplementary material for: A loss-of-function variant in ZCWPW1 causes human male infertility with sperm head defect and high DNA fragmentation
Source: Reprod Health. 2024 Feb 3;21:18. doi: 10.1186/s12978-024-01746-9 (PMC10837985; doi:10.1186/s12978-024-01746-9)
Supplement: Supplementary file 1 — Additional file 1. Figure S1. The localization of mutant ZCWPW1 did not alter in vitro. We used anti-Flag antibofy to detected exogenous ZCWPW1 in MUT-ZCWPW1 transfected cells compared to WT-ZCWPW1 transfected cells. Effectively transfected cells were marked with arrowhead. (Scale bars, 10μm). Figure S2. The reduced expression of ZCWPW1 in proband’s sperms. The relative fluorescence intensity analysis normalized by α-Tubulin was shown. Data represent the mean ± SD from three independent experiments. Student’s t-test, **P < 0.01. [file 12978_2024_1746_MOESM1_ESM.docx]

**Supplementary material**

**Supplementary Figure 1**


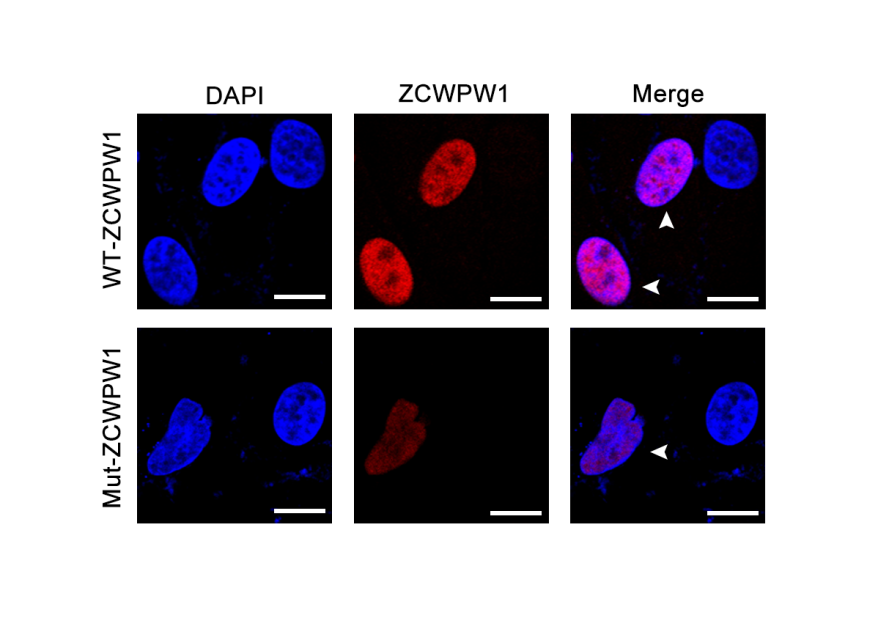


The localization of mutant ZCWPW1 did not alter in vitro. We used anti-Flag antibofy to detected exogenous ZCWPW1 in MUT-ZCWPW1 transfected cells compared to WT-ZCWPW1 transfected cells. Effectively transfected cells were marked with arrowhead.(Scale bars, 10μm).

**Supplementary Figure 2**

**
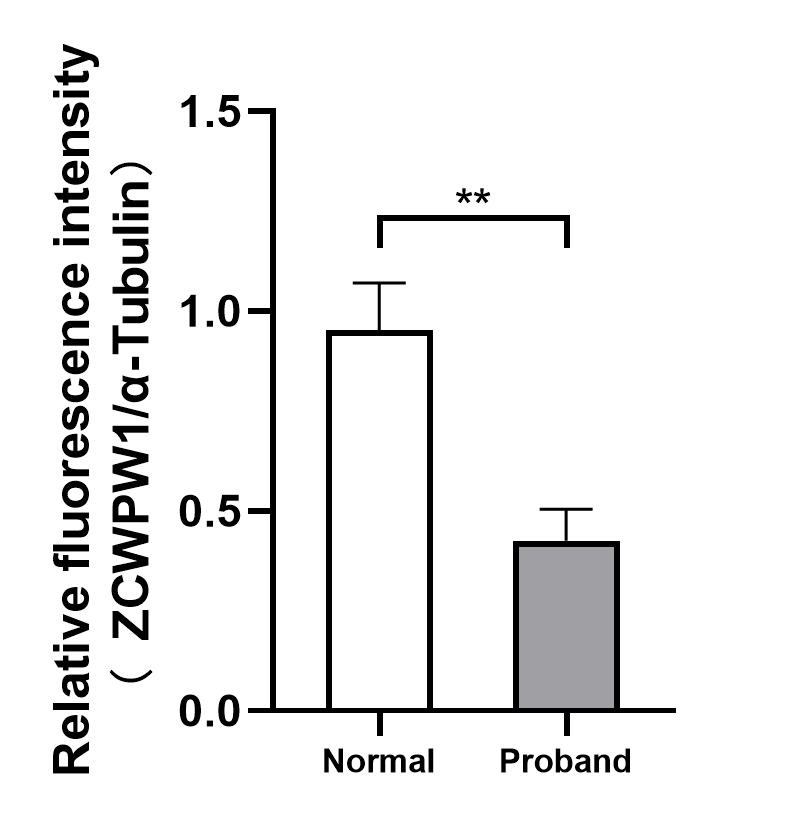
**

The reduced expression of ZCWPW1 in proband's sperms. The relative fluorescence intensity analysis normalized by α-Tubulin was shown. Data represent the mean ± SD from three independent experiments. Student’s t-test, **P < 0.01.
